# Dr. Charles W. West

**Published:** 1860-09

**Authors:** 


DR. CHARLES W. WEST expired on the fourth of June, in the forty-fifth year of his age, in consequence of injuries received from being thrown from his buggy. Dr. West received his medical degree at the Augusta Medical College; and soon after the completion of his studies sailed for Paris, where he remained more than two years. Upon his arrival in Georgia, he accepted the professorship of Chemistry, in the Augusta school, but on account of ill health was obliged to vacate his chair. Dr. West for several years lectured on Chemistry in the Savannah Medical College, an institution of which he was one of the founders.

